# Multiple spatial scale mapping of time-resolved brain network reconfiguration during evoked pain in patients with rheumatoid arthritis

**DOI:** 10.3389/fnins.2022.942136

**Published:** 2022-08-09

**Authors:** Silvia Fanton, Reem Altawil, Isabel Ellerbrock, Jon Lampa, Eva Kosek, Peter Fransson, William H. Thompson

**Affiliations:** ^1^Department of Clinical Neuroscience, Karolinska Institutet, Stockholm, Sweden; ^2^Department of Neuroradiology, Karolinska University Hospital, Stockholm, Sweden; ^3^Rheumatology Unit, Department of Medicine, Center for Molecular Medicine, Karolinska Institutet, Karolinska University Hospital, Stockholm, Sweden; ^4^Department of Surgical Sciences, Uppsala University, Uppsala, Sweden; ^5^Division of Cognition and Communication, Department of Applied IT, University of Gothenburg, Gothenburg, Sweden

**Keywords:** fMRI, brain networks, time-varying functional connectivity, temporal network theory, chronic pain, rheumatoid arthritis

## Abstract

Functional brain networks and the perception of pain can fluctuate over time. However, how the time-dependent reconfiguration of functional brain networks contributes to chronic pain remains largely unexplained. Here, we explored time-varying changes in brain network integration and segregation during pain over a disease-affected area (joint) compared to a neutral site (thumbnail) in 28 patients with rheumatoid arthritis (RA) in comparison with 22 healthy controls (HC). During functional magnetic resonance imaging, all subjects received individually calibrated pain pressures corresponding to visual analog scale 50 mm at joint and thumbnail. We implemented a novel approach to track changes of task-based network connectivity over time. Within this framework, we quantified measures of integration (participation coefficient, PC) and segregation (within-module degree *z*-score). Using these network measures at multiple spatial scales, both at the level of single nodes (brain regions) and communities (clusters of nodes), we found that PC at the community level was generally higher in RA patients compared to HC during and after painful pressure over the inflamed joint and corresponding site in HC. This shows that all brain communities integrate more in RA patients than in HC for time points following painful stimulation to a disease-relevant body site. However, the elevated community-related integration seen in patients appeared to not pertain uniquely to painful stimulation at the inflamed joint, but also at the neutral thumbnail, as integration and segregation at the community level did not differ across body sites in patients. Moreover, there was no specific nodal contribution to brain network integration or segregation. Altogether, our findings indicate widespread and persistent changes in network interaction in RA patients compared to HC in response to painful stimulation.

## Introduction

Alterations in the functional connectivity between brain regions have been reported in patients with chronic pain (Napadow et al., 2010; Tagliazucchi et al., 2010; Hemington et al., 2016), bringing factual contribution to the consideration of chronic pain as a condition that can be studied and understood from a brain network modeling perspective (Apkarian et al., 2009; Mano et al., 2018).

Within this framework, recent advances have been made in the identification of an objective biomarker of chronic pain. Notably, a recent study provided evidence for a neuroimaging marker for tonic experimental pain predicting sustained clinical pain (Lee et al., 2021). An interesting feature of this biomarker signature is its largely distributed network-level representation of the sustained pain state (Lee et al., 2021). Yet recently, the organization of networks in the brain was proposed as potential biomarker and further investigated, specifically, as the assignment of nodes (brain regions) to different communities (clusters of nodes) in the whole-network (brain) (Larkin et al., 2021) and via the examination of brain hub topology (Kaplan et al., 2019). Interestingly, the hub topology was altered (Kaplan et al., 2019) and the allocation of nodes in communities more variable (Larkin et al., 2021) in chronic pain patients compared to healthy controls (HC), providing knowledge into both the local and global functional resting-state network architecture of chronic pain patients (Larkin et al., 2021).

Variables obtained from modeling functional magnetic resonance imaging (fMRI) data in the context of time-varying brain networks may act as more sensitive markers of acute and chronic pain, given the dynamic nature of pain and the brain itself. Specifically, there is evidence to suggest that the organization of brain networks fluctuates between states of integration and segregation (Shine et al., 2016) and, within a time-varying functional connectivity (TVC) framework, these measures have been proven to be critical in understanding cognition (Cohen and D’Esposito, 2016; Shine et al., 2016; Fransson et al., 2018).

However, the application of integration and segregation measures to the investigation of pain-related patterns of network reconfiguration is still in its infancy. Recent work from our group assessed TVC changes in network integration/segregation in HC during thermal pain, showing increased brain network integration with increased pain (Kastrati et al., 2022). To add specificity to the investigation of pain processing in chronic pain patients, we used TVC to explore changes in brain network integration and segregation that are time-locked to pressure pain stimulations over a disease-affected body site (i.e., inflamed joint) and a neutral body part (i.e., thumbnail) in chronic pain patients with rheumatoid arthritis (RA) compared to HC. Notably, pain pressure stimuli were individually calibrated across both groups and sites. When comparing the same cohort of RA patients and HC, previous work from our group showed: (1) increased intrinsic, static FC between bilateral sensorimotor and frontal midline brain regions in patients compared to HC (Flodin et al., 2016), (2) reduced activation in brain regions associated to the processing of pain and somatosensory information in patients compared to HC when painful stimulation is delivered to the joint, and not to the thumb (Sandström et al., 2019). When comparing body sites within patients, Sandström et al. (2019) showed that abnormalities in cerebral pain processing in patients were confined uniquely to the joint (i.e., the disease-affected site) and not generalizable to the thumb (i.e., the neutral area), with patients exhibiting a reduced activation in somatosensory and pain processing regions as well as in coupled right and left dorsolateral prefrontal cortex.

Based on these premises, the objective of the present work was to determine whether and how the degree of change in integration and segregation between network communities and nodes varies over time and is putatively influenced by pressure pain stimuli across groups and stimulation sites in patients. This might add more specificity to the understanding of cerebral pain processing mechanisms in patients with RA.

## Materials and methods

### Participants and study description

The dataset used in the current study has previously been described in Flodin et al. (2016) and in Sandström et al. (2019). A detailed account of participants and information regarding exclusion and inclusion criteria can be found in Sandström et al. (2019). Participants underwent two testing sessions, on two consecutive days. Of relevance to the current study, on day 1, sensitivity to evoked pressure pain was individually calibrated and, on day 2, the individually calibrated painful pressure and a non-painful pressure were delivered during four runs of a functional magnetic resonance imaging (fMRI) pressure pain paradigm. fMRI data (covering the whole brain) from a total of 28 RA patients and 22 HC were included in the analysis (mean age RA patients = 53.64 years; mean age HC = 52.86 years; age range = 23–72 years). The minor difference in the total number of subjects included in this study compared to Sandström et al. (2019) is due to the need to adhere to a specific pipeline tailored to time-resolved fMRI data. All participants gave written informed consent in accordance with the Declaration of Helsinki. The local ethical review board approved the research.

### Experimental procedure

The present study forms part of a larger project (referred to as the PARADE study; https://www.clinicaltrials.gov; [identifier NCT01197144, EudraCT 2009-017163-42]). Previously, we have reported differences in spontaneous brain activation patterns (Flodin et al., 2016) and brain activity recorded after painful stimuli delivered to a disease-affected finger joint as well as to the non-affected thumbnail area (Sandström et al., 2019). In this work, we use time-varying functional connectivity (TVC), which allows for a time point-by-time point assessment of changes in brain network activity related to pain. Therefore, the methodological section in this paper is focused on the procedure used for assessing TVC.

#### Day 1: Individual calibration of pressure pain

One day prior to fMRI scanning, the degree of pain pressures to be used during scanning was subjectively calibrated. Pain sensitivity was assessed by applying pressure to the patient’s clinically most affected proximal interphalangeal (PIP) joint (PIP2 *n* = 21; PIP3 *n* = 7) of the left hand and to the non-affected, left thumbnail via an automated, pneumatic, computer-controlled stimulator with a 1 cm^2^-hard rubber probe (Jensen et al., 2009). Corresponding anatomical sites were used in HC (PIP2 *n* = 21; PIP3 *n* = 1). Each participant first received a series of stimuli with a step-wise increase in pressure, then followed by a series of stimuli that had a randomized order of different pressure. The pressure stimuli, in both series, were delivered for a duration of 2.5 s and with 30 s inter-stimulus intervals. After each stimulus, participants were prompted to rate pain intensity on a 0–100 mm visual analog scale (VAS), ranging from “no pain” to “worst imaginable pain.” Stimuli in the ascending series were presented in increasing pressure steps of 50 kPa, which led to the identification of each participant’s pressure pain threshold (PPT, first VAS rating > 0 mm) and stimulation maximum (SM, first VAS rating > 60 mm). It is within this subjectively calibrated range of PPT and SM that five pressure pain intensities were obtained and delivered, each three times, in a randomized series. A polynomial regression was applied to the 15 VAS ratings from the randomized series and, consequently, used to determine each subjective representation of VAS 50 mm (referred to as P50). Please refer to Jensen et al. (2009) for further details regarding the calibration procedure.

#### Day 2: Functional magnetic resonance imaging pressure pain paradigm

The subjectively calibrated painful pressure (P50) obtained from day 1 and a standard non-painful pressure (50 kPA) were presented during four pseudo-randomized fMRI runs. Two of the four fMRI runs contained stimuli that were delivered to the most affected joint in patients (equivalent anatomical site in HC), while the remaining two fMRI runs included stimuli applied to the thumbnail. Each run consisted of a total of 30 pressure stimuli events (15 painful and 15 non-painful) presented in a pseudo-randomized fashion. The duration of each stimulus was 2.5 s and all stimuli onsets were jittered over time with a mean interval of 15 s (range 10–20 s) to ensure a fine-grained sampling of the events. The total duration for all runs was 8 min and 15 s. All participants were instructed, prior to scanning, to concentrate on the pressures delivered to joint and thumbnail and to refrain from invoking coping strategies. No pain ratings were collected during the course of the fMRI runs.

### Functional magnetic resonance imaging data acquisition and pre-processing

MRI data were acquired on a 3T General Electric 750 MR scanner installed at the MR Research Center at Karolinska Institutet (Stockholm) using a 32-channel head coil. Four task-based fMRI scans, each consisting of 160 volumes, were acquired for each subject using a T2*-weighted single-shot gradient echo planar sequence (TR/TE = 3000/30 ms; 90° flip angle; 96 × 96 matrix size; FOV = 288 × 288 mm; 56 slices; in-plane resolution = 2.5 × 2.5 mm; slice thickness = 3 mm, interleaved slice acquisition). Anatomical MRI data were obtained using a high-resolution T1-weighted image sequence (3D BRAVO; TR/TE = 7908/3.06 ms; 1 × 1 × 1 mm voxel size; 176 slices). Additionally, T2-weighted images were acquired and assessed for clinical abnormalities by a neuroradiologist.

The pre-processing of anatomical and functional data was performed using fMRIPrep (pre-processing pipeline, version 20.1.1, Esteban et al., 2019). A detailed description of all the pre-processing steps can be found in the Supplementary material.

### Analysis pipeline for time-varying functional connectivity

**FIGURE 1:**
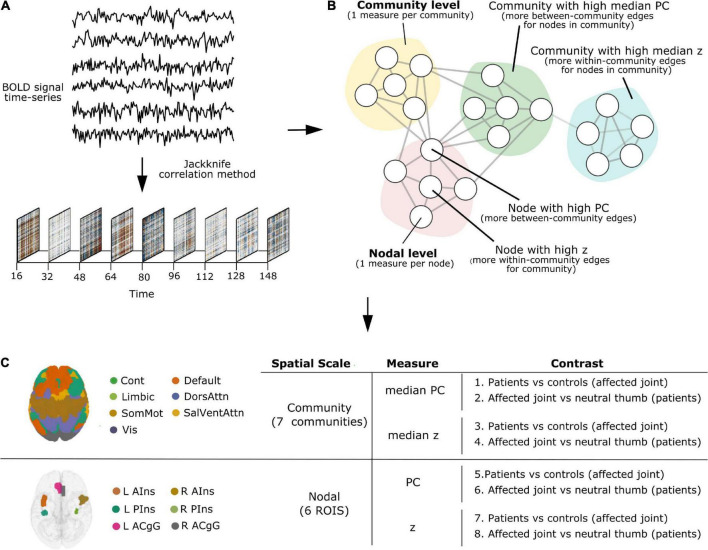
A schematic representation of the time-varying functional connectivity (TVC) analysis steps undertaken in this work. **(A)** BOLD signal time-series were extracted from brain regions defined using the 7-network, 400-node Schaefer parcellation scheme (Yeo et al., 2011; Schaefer et al., 2018) and TVC was computed at the level of single time points using the jackknife correlation method (Richter et al., 2015). **(B)** Measures of integration (participation coefficient, PC) and segregation (within-module degree *z*-score, *z*) were quantified and used at the level of single nodes (brain areas, nodal level) and communities (clusters of nodes, community level). **(C)** Analyses were carried out at the community and nodal level, separately. Within each level, we examined how the degree of integration (PC) and segregation (*z*) varies over time and whether this is influenced by painful stimulation to the joint across groups (Contrasts 1, 3, 5, 7) and based on the stimulation site in patients (Contrasts 2, 4, 6, 8).

All TVC analyses reported below (parcellation, denoising, deriving TVC estimates, quantifying network measures of participation coefficient and within-module degree z-score) were carried out using teneto (version 0.5.3), a Python package for temporal network analysis (Thompson et al., 2017a)^1^. Please refer to Figure 1 for a schematic representation of the TVC analysis steps undertaken in this work. The code used to set up the teneto pipeline and to produce figures is available at https://github.com/silviafan/TVC-RA.

### Parcellation and functional magnetic resonance imaging data denoising

The BOLD signal time-series from the pre-processed fMRI data were extracted from brain areas defined using the 7-network (community), 400-node Schaefer parcellation scheme (Yeo et al., 2011; Schaefer et al., 2018). Subsequent to the parcellation step, functional fMRI data underwent denoising, with the following confounds being regressed out: six head motion parameters and their respective derivatives, the first six noise anatomical parameters derived from CompCor (Behzadi et al., 2007), framewise displacement (FD, Power et al., 2014), white matter, and cerebrospinal fluid. Additionally, we employed the criteria that fMRI runs that had a mean FD > 0.5 were to be discarded from the analyses. No fMRI run met this exclusion criteria.

#### Deriving parameter estimates for time-varying functional connectivity

The time-varying changes in functional connectivity were estimated via the application of the jackknife correlation (JC) method (Richter et al., 2015). When applied to TVC, the JC method calculates the Pearson’s correlations between two BOLD time-series *x* and *y* over all the individual time points, excluding data at time point *t*, and then multiplies the resulting correlation value by –1 (Thompson et al., 2018).


$$J\,C_{t}=-\left(\frac{\sum_{i}^{T}\left(x_{i-}\,{\bar{x}}_{t}\right)\,\left(y_{i-}\,{\bar{y}}_{t}\right)}{\sum_{i}^{T}{\left(x_{i-}\,{\bar{x}}_{t}\right)}^{2}\,\sum_{i}^{T}{\left(y_{i-}\,{\bar{y}}_{t}\right)}^{2}}\right)\;i\neq t$$

This application of the JC method to compute time-varying functional connectivity was first introduced by Thompson et al. (2017b). Importantly, it has been previously shown that the JC method is more sensitive to quicker temporal changes in covariance compared to other methods (Thompson et al., 2018; Xie et al., 2019). Here, JC values were standardized to have mean of 0 and standard deviation of 1 creating flow TVC estimates (Fransson and Thompson, 2020). Note that the JC method provides time point-by-time point estimates of pairwise functional brain connectivity.

#### Quantifying time-varying parameters of community integration and segregation

To answer the question of how the degree of integration and segregation of the seven pre-defined communities and six chosen nodes changes over time and is putatively influenced by painful stimulation to the joint across groups and based on the stimulation site, we chose to include network measures that have been previously shown to be informative and relevant for other *qualia* (Cohen and D’Esposito, 2016; Shine et al., 2016), although never before used in the context of chronic pain.

First, we calculated the participation coefficient (PC), a network metrics that quantifies the degree to which specific nodes communicate across communities (Guimerà and Nunes Amaral, 2005). As described in Guimerà and Nunes Amaral (2005), PC is:


$$P_{i}=1-\sum_{s=1}^{N_{M}}{\left(\frac{k_{i\,s}}{k_{i}}\right)}^{2}$$

Where *i* = node, *s* = index of community (*N*_*M*_), *k_*is*_* = degree of links within each community, *k_*i*_* = total degree of *i*. In the computation of PC values, only positive edges were included in the analysis. Next, we computed the within-module degree *z*-score (z), a network metrics that measures the extent to which specific nodes communicate within their own communities (Guimerà and Nunes Amaral, 2005). As described in Guimerà and Nunes Amaral (2005), *z* is:


$$z_{i}=\frac{k_{i}-{\bar{k}}_{s_{i}}}{\sigma_{k_{s_{i}}}}$$

Where i = node, *k_*i*_* = degree of links of *i* to other nodes in its community *s*_*i*_, ${\bar{k}}_{s_{i}}$ = average of k across nodes in community *s*_*i*,_ σ_*k_s_i__*_ = standard deviation of k in community *s*_*i*_.

For all analyses, we used the same static parcellation for all time points (Thompson et al., 2020). Taken together, these two measures provide insights into the integration (PC) and segregation (*z*) of nodes in the whole network.

After computing PC and *z* for all nodes, integration and segregation were assessed both at community level (i.e., measures per community) and nodal level (i.e., measures per node). The idea was to probe brain network organization at multiple spatial scales (Sporns, 2015), by capturing the temporal dynamics of pain processing in the whole-brain (community level) and at the level of single pain-related brain regions (nodal level).

On the community level, the median PC or *z* of all nodes within each community was calculated. Note, while the mean of *z* is always 0 for each community, the median denotes if the majority of edges are skewed below or above the mean, making the different community distributions comparable regarding how the community as a whole is tightly connected. Thus, the median PC or *z* can be interpreted as the degree of *integration* or *segregation* a community as a whole has with respect to the other communities in the whole network. For example, as illustrated in Figure 1B, a community with high median PC has more *between-community* edges for nodes in the community (*integration* on the community level), whereas a community with high median *z* has more *within-community* edges for nodes in the community (*segregation* on the community level).

**FIGURE 2:**
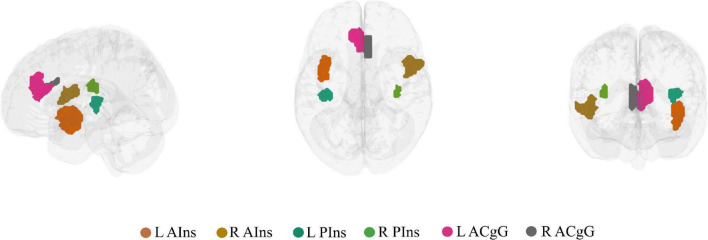
The six brain nodes selected to represent key chronic pain brain regions shown in a lateral, superior, and anterior brain view. L AIns, left anterior insula; R AIns, right anterior insula; L PIns, left posterior insula; R PIns, right posterior insula; L ACgG, left anterior cingulate gyrus; R ACgG, right anterior cingulate gyrus.

On the nodal level, node selection was informed by overlapping (performed in MRIcron^2^) the brain activation map produced by entering the text query “chronic pain” into NeuroQuery, a recently developed tool for comprehensive meta-analysis of the neuroimaging literature (Dockès et al., 2020), onto the 7-network, 400-node Schaefer parcellation (Yeo et al., 2011; Schaefer et al., 2018). This procedure resulted in the selection of six brain areas (nodes): left anterior insula (L AIns), right anterior insula (R AIns), left posterior insula (L PIns), right posterior insula (R PIns), L anterior cingulate gyrus (L ACgG), and right anterior cingulate gyrus (R ACgG). Please refer to Figure 2 for a representation of the selected nodes and to Supplementary Table 1 for their identification by templateflow index (Ciric et al., 2021). Obviously, this approach necessitated some degree of subjectivity in terms of the selection of nodes to include but was, nevertheless, undertaken for the purpose of all results (community and nodal level-related results) to be aligned within the same whole-brain parcellation. The PC or z score for each single node can be defined as the degree of *integration* or *segregation* a node has with respect to the other nodes in the whole network. For example, as illustrated in Figure 1B, a node with high PC has more *between-community* edges (*integration* on the nodal level), whereas a node with high z has more *within-community* edges for the community (*segregation* on the nodal level).

In order to account for the temporal profile of brain connectivity across the two spatial scales (community and nodal), the 160 time point data series were broken down into 6 event-related bins, with bins representing the onset TR when the stimulation was being delivered and until –2 TR pre- and + 3 TR after-stimulus onset, each averaged across participants. This binning partition served as an indicator for the time-varying changes in functional connectivity occurring before, during, and after participants received the painful stimulation.

### Statistical analyses

All analyses were carried out using Python version 3.7.2 and were performed: (1) separately for each community and nodal level, (2) only for painful stimuli, (3) by averaging together the two joint or the two thumb runs, respectively, and (4) by focusing only on onset and after-onset time points, each treated separately. Pre-onset time points were not included in the analyses, as the pain pressure task used in the study had not been designed to capture the cerebral processing involved in anticipating pain. However, these pre-onset time points were nonetheless plotted in the graphs, for completeness. Statistical significance was set, conventionally, at *p* < 0.05, false discovery rate (FDR) corrected for multiple comparisons.

#### Group differences in integration and segregation at nodal and community level for painful stimulation of the joint

A one-way analysis of covariance (ANCOVA) test was performed using the *ols* function in the Python *statsmodels* library (Seabold and Perktold, 2010) to test for differences across groups in the degree of change in integration and segregation over time at the community and nodal level in response to painful stimulation of the affected joint in patients compared to the corresponding site in HC (Contrasts 1, 3, 5, and 7 in Figure 1C). In the model, the network metric under investigation (PC, *z*) was used as dependent variable, the group variable was treated as independent variable with two levels (RA, HC), and age was used as a covariate. Despite the unbalanced design, Type II Sum of Squares was used, as proposed by Langsrud (2003).

#### Differences in integration and segregation at nodal and community level for painful stimulation of the joint compared to thumb in patients

Differences in the degree of change in integration and segregation over time at the community and nodal level in response to painful stimulation of the affected joint compared to the neutral thumb in patients (Contrasts 2, 4, 6, and 8 in Figure 1C) were tested by performing a linear mixed-effects model with the *statsmodels* implementation (MixedLM). The variable “site” (joint, thumb) was entered into the model as fixed effect while controlling for age. A random intercept for each subject and, by default, for each site (joint, thumb) were also introduced to account for the variability of subjects and sites (joint, thumb) at baseline. The model was adjusted, also by default, by the Restricted Maximum Likelihood Estimation and the Powell optimization method was used for model fitting.

Brain plots were generated using *netplotbrain* (Thompson and Fanton, 2021^3^).

## Results

### Painful stimulation of the joint induces higher community-wide integration, but no difference in segregation, in patients compared to controls

**FIGURE 3:**
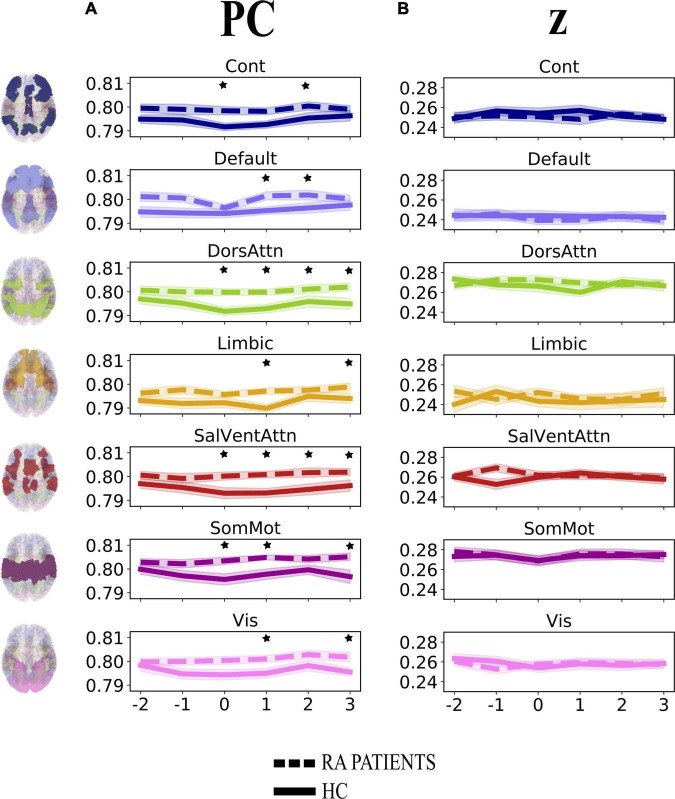
Degree of change in community-related integration (A) and segregation (B) per time point for rheumatoid arthritis (RA) patients and healthy controls (HC), when pain was delivered to the diseased joint and corresponding site in HC. Displayed are the average parameter values (PC, *z*) over trials per time point. Time points are indicated as –2, –1, 0, +1, +2, +3 on the *x*-axis, with 0 representing the onset TR (TR = 3 s) of painful stimulation, –2 and –1 being the two TR pre-stimulation, whereas + 1, +2, and +3 the three TR after-stimulation. The brain plots on the left side of the figure represent each of the seven Yeo communities (Yeo et al., 2011). The shaded areas contouring the lines represent the standard error of the mean. The stars represent time points that differed significantly (*p* < 0.05, FDR corrected) between groups. PC, participation coefficient; *z*, within-module degree *z*-score; RA, rheumatoid arthritis; HC, healthy controls.

First, we investigated the degree of change over time in integration (participation coefficient, PC) and segregation (within-module degree *z*-score, *z*) at the community level across groups when pain was delivered to the diseased joint and corresponding site in HC (Contrasts 1 and 3 in Figure 1C). Here, PC was found to be generally higher in patients, compared to HC, in all brain communities and for some time points (Table 1 and Figure 3A). However, groups (patients and HC) did not differ in the degree of community-related segregation (*z*) change over time (Figure 3B and Supplementary Table 2).

**TABLE 1:** Results from the analysis of covariance (ANCOVA) computed at the community level on participation coefficient (PC) values per time point across groups (patients and controls) when pain was delivered to the diseased joint in patients and corresponding site in controls.

PC												
	0			+1			+2			+3		
	*F*	*p*	FDR p	*F*	*p*	FDR p	*F*	*p*	FDR p	*F*	*p*	FDR p
	
Cont	7.218	0.010	**0.038**	2.997	0.090	0.120	5.157	0.028	**0.043**	1.760	1.191	0.216
Default	0.567	0.455	0.455	5.047	0.029	**0.043**	5.188	0.027	**0.043**	1.084	0.303	0.317
DorsAttn	9.614	0.003	**0.023**	5.246	0.027	**0.043**	6.192	0.016	**0.038**	11.281	0.002	**0.022**
Limbic	1.742	0.194	0.216	6.169	0.017	**0.038**	1.071	0.306	0.317	5.207	0.027	**0.043**
SalVentAttn	8.593	0.005	**0.029**	6.261	0.016	**0.038**	6.627	0.013	**0.038**	6.139	0.017	**0.038**
SomMot	10.007	0.003	**0.023**	6.057	0.018	**0.038**	2.688	0.108	0.131	13.114	0.001	**0.020**
Vis	4.215	0.046	0.064	5.254	0.026	**0.043**	2.756	0.104	0.131	6.545	0.014	**0.038**

Time points are indicated as 0, +1, +2, +3 and represent, respectively, the onset TR (TR = 3 s) of painful stimulation, and +1, +2, and +3 TR after-stimulation. False discovery rate (FDR) corrected p-values, significant at the conventional p < 0.05, are presented in bold.

### No significant group difference in node-related integration or segregation for painful stimulation of the joint

Next, we analyzed the degree of change over time in integration (participation coefficient, PC) and segregation (within-module degree *z*-score, *z*) at the nodal level across groups when pain was delivered to the diseased joint and corresponding site in HC (Contrasts 5 and 7 in Figure 1C). Contrarily to integration at the community level, groups did not differ in node-related integration (Supplementary Figure 1A and Supplementary Table 3) nor segregation (Supplementary Figure 1B and Supplementary Table 4) over time.

### No significant difference in integration and segregation at nodal and community level due to the different stimulation site (joint vs. thumb) in patients

Lastly, we examined the degree of change over time in integration (participation coefficient, PC) and segregation (within-module degree *z*-score, *z*) at the community and nodal level in patients when pain was delivered to the diseased joint compared to the neutral thumb (Contrasts 2, 4, 6, and 8). None of the analyses showed any significant degree of change (Supplementary Figures 2, 3 and Supplementary Tables 5–8).

## Discussion

The present study capitalized on previously analyzed fMRI data in order to detail the temporal profile of cerebral pain processing in patients with RA, with the final objective to track pain-related patterns of network reconfiguration at the resolution of single data time points.

A major finding of this work is that the participation coefficient was generally higher in RA patients compared to HC during and following pressure pain over the inflamed joint compared to the corresponding site in HC. This result shows that all brain communities integrate to a relatively higher degree in patients than in HC during some, but not all, time points when painful stimulation is delivered to the disease-relevant body site (Figure 3A). Interestingly, our finding of increased community-level integration is in accordance with previous research on pain and cognition, with the latter being a critical component contributing to the multi-dimensionality of pain experience. Regarding pain research, recent work has shown that: (1) in HC, there is elevated integration of brain networks in the presence of more intense pain (Kastrati et al., 2022), (2) a tonic pain model, capable of predicting experimental and clinical sustained low back pain, provides evidence for the involvement of a number of highly distributed brain networks during a sustained pain state (Lee et al., 2021), and (3) the experience of pain is supported by comprehensive multi-network interactions (Geuter et al., 2020). Regarding the cognitive aspect, several studies have shown an increase in or shift to a state of higher integration between brain networks during the execution of demanding cognitive tasks (Cohen and D’Esposito, 2016; Fransson et al., 2018). That being said, in our work, the observation of an up-ramped degree of integration among the canonical resting-state networks as defined in the Schaefer atlas during and after painful stimulation of the clinically affected area might be responsible for an overly energetically demanding experience for the brain. Indeed, although the brain has long been regarded as a complex system where integration plays a key and decisive role (Tononi, 1998), interactions between communities are costly to maintain, thus occur in alternation with more highly modular periods (Liégeois et al., 2016). Importantly, Shine et al. (2016) have shown that states of integration allow for a more effective and faster processing of information during task execution. However, with reference to our finding and on a speculative note, in the presence of painful information, a sustained state of whole-brain integration, without intermitting segregation, might accelerate the propagation of recursive noxious information. Consequently, this might lead to the exacerbation of pain experience and, in turn, contribute to pain chronicity in patients. Further, though this state of higher integration in patients compared to HC concerns all brain communities, it distributes differently over time (see Table 1 and Figure 3A). Indeed, the higher integration seen in patients features onset and all after-onset time points in, specifically, DorsAttn and SalVentAttn communities. As for the other tested resting-state networks, group differences were less “durable.” A plausible, although speculative, explanation might be that the salience network seems to be relevant in regulating the functional changes of other networks dynamically (Bonnelle et al., 2012), as also pointed out in Borsook et al. (2013), and has been shown to be associated with attention networks, given the dependency between saliency and attention (Menon and Uddin, 2010; Uddin, 2015). Thus, on a speculative level, it might be that the constant high integration of the SalVentAttn and DorsAttn communities is coordinating the functional role of the other communities, thus reducing their integration at times to favor the required high-level attention to salient, painful stimuli. This finding might indicate that the brain of patients enters a state of persistent high integration, not allowing the brain to be in a more modular state at any time. Possibly, this might be a contributing factor to the cognitive fatigue affecting chronic pain patients and, relevant to this work, patients with RA (Nikolaus et al., 2013).

When exploring whether this maladaptive brain network configuration in patients was specific to when pain was delivered to the inflamed joint or also to the non-clinically relevant thumb, our results pointed to the latter. Indeed, there was no difference in community-related integration over time across body sites (Supplementary Figure 2A), which might be interpreted as elevated integration not uniquely pertaining to the inflamed area, but also to an area that appears “neutral” when the peripheral nervous system is the focus. Thus, this might be indicative of patients having a more generalized and unspecific cerebral response to pain, unbound to the clinical relevance of the stimulation site. This finding seems to be in conflict, at first, with previous work published from our group, in which it was shown that patients had lower pain-related cerebral activation in response to painful stimulation at the joint compared to the thumb (Sandström et al., 2019). However, it is not surprising that a finding reporting task-evoked BOLD response lacks direct translation into a task-based TVC context. Indeed, although specific to the default mode network and its regions, it has been previously demonstrated that, for example, task-related negative BOLD signal does not affect the temporal profile of task-related FC networks (Razlighi, 2018), thus in accordance with the disagreement in our previous (Sandström et al., 2019) and current results.

Shifting focus from the organization of communities to the role of single nodes in the whole network, all our results indicate that there is no contribution from the six selected network nodes to brain network integration or segregation, neither during pain to joint across groups, nor during pain to joint compared to thumb within patients only (Supplementary Figures 1, 3). This might indicate that the community level may be more informative than the nodal level in terms of revealing potential differences in cerebral pain processing between patients and HC. However, the lack of significant results at the nodal level might be due to the fact that the *robustness* of the measures applied at the nodal level might have been more easily affected by the high number of multiple comparisons. While for PC and *z* at the community level, we computed the median of all nodes within each community, for PC and *z* at the nodal level, we considered single nodes among the 400 nodes generated by the Schaefer 400 node x 7 network parcellation. Further, we note that, as also discussed by Kastrati et al. (2022), brain communities were regarded as static clusters of nodes and their integration and segregation profile was investigated over time. Thus, allowing nodes to be dynamically assigned to different communities via community detection might further inform about local reconfigurations and their contribution to the functional architecture of the network in a pain context, as mentioned in Kastrati et al. (2022).

The balance between integration and segregation is crucial for the brain (Shine, 2019). Whereas higher thermal pain has been shown to already disrupt this balance in HC inducing a shift from segregation to integration (Kastrati et al., 2022), in patients with RA, we might speculatively argue that, based on our results, this balance appears to be undergoing a constant perturbation in favor of a permanent high integration state.

With the present study, we were able to track the temporal profile of pain-related network changes in RA patients and HC. From a clinical perspective, this is of great importance for the understanding of the mechanisms involved in the perception of pain and could possibly contribute to the identification of a brain-based biomarker for chronic pain.

## Data availability statement

The original contributions presented in this study are included in the article/Supplementary material, further inquiries can be directed to the corresponding author.

## Ethics statement

The studies involving human participants were reviewed and approved by the regional ethical committee of Stockholm. The patients/participants provided their written informed consent to participate in this study.

## Author contributions

SF: conceptualization, methodology, formal analysis, visualization, and writing – original draft and review and editing. RA: data curation, investigation, and writing – review and editing. IE: contribution to formal analysis and writing – review and editing. JL: data curation, funding acquisition, investigation, project administration, resources, and writing – review and editing. EK: conceptualization, data curation, funding acquisition, investigation, project administration, resources, supervision, and writing – review and editing. PF: supervision and writing – review and editing. WT: conceptualization, methodology, supervision, contribution to formal analysis, contribution to visualization, and writing – review and editing. All authors contributed to the article and approved the submitted version.

## Footnotes

1
https://github.com/wiheto/teneto

2
https://www.nitrc.org/projects/mricron

3
https://github.com/wiheto/netplotbrain

## References

[B1] Apkarian A. V. Baliki M. N. Geha P. Y. (2009). Towards a theory of chronic pain. *Prog. Neurobiol.* 87 81–97. 10.1016/j.pneurobio.2008.09.018 18952143 PMC2650821

[B2] Behzadi Y. Restom K. Liau J. Liu T. T. (2007). A component based noise correction method (CompCor) for bold and perfusion based fMRI. *NeuroImage* 37 90–101. 10.1016/j.neuroimage.2007.04.042 17560126 PMC2214855

[B3] Bonnelle V. Ham T. E. Leech R. Kinnunen K. M. Mehta M. A. Greenwood R. J. (2012). Salience network integrity predicts default mode network function after traumatic brain injury. *Proc. Natl. Acad. Sci.* 109 4690–4695. 10.1073/pnas.1113455109 22393019 PMC3311356

[B4] Borsook D. Edwards R. Elman I. Becerra L. Levine J. (2013). Pain and analgesia: the value of salience circuits. *Prog. Neurobiol.* 104 93–105. 10.1016/j.pneurobio.2013.02.003 23499729 PMC3644802

[B5] Ciric R. Thompson W. H. Lorenz R. Goncalves M. MacNicol E. Markiewicz C. J. (2021). TemplateFlow: fAIR-sharing of multi-scale, multi-species brain models. *Cold Spring Harb. Lab.*[Preprint] 10.1101/2021.02.10.430678 PMC9718663 36456786

[B6] Cohen J. R. D’Esposito M. (2016). The segregation and integration of distinct brain networks and their relationship to cognition. *J. Neurosci.* 36 12083–12094. 10.1523/JNEUROSCI.2965-15.2016 27903719 PMC5148214

[B7] Dockès J. Poldrack R. A. Primet R. Gözükan H. Yarkoni T. Suchanek F. (2020). NeuroQuery, comprehensive meta-analysis of human brain mapping. *ELife* 9:e53385. 10.7554/eLife.53385 32129761 PMC7164961

[B8] Esteban O. Markiewicz C. J. Blair R. W. Moodie C. A. Isik A. I. Erramuzpe A. (2019). fMRIPrep: a robust preprocessing pipeline for functional MRI. *Nat. Methods* 16 111–116. 10.1038/s41592-018-0235-4 30532080 PMC6319393

[B9] Flodin P. Martinsen S. Altawil R. Waldheim E. Lampa J. Kosek E. (2016). Intrinsic brain connectivity in chronic pain: a resting-state fMRI study in patients with rheumatoid arthritis. *Front. Hum. Neurosci.* 10:107. 10.3389/fnhum.2016.00107 27014038 PMC4791375

[B10] Fransson P. Schiffler B. C. Thompson W. H. (2018). Brain network segregation and integration during an epoch-related working memory fMRI experiment. *NeuroImage* 178 147–161. 10.1016/j.neuroimage.2018.05.040 29777824

[B11] Fransson P. Thompson W. H. (2020). Temporal flow of hubs and connectivity in the human brain. *NeuroImage* 223 117348. 10.1016/j.neuroimage.2020.117348 32898675

[B12] Geuter S. Reynolds Losin E. A. Roy M. Atlas L. Y. Schmidt L. Krishnan A. (2020). Multiple brain networks mediating stimulus–pain relationships in humans. *Cereb. Cortex* 30 4204–4219. 10.1093/cercor/bhaa048 32219311 PMC7264706

[B13] Guimerà R. Nunes Amaral L. A. (2005). Functional cartography of complex metabolic networks. *Nature* 433 895–900. 10.1038/nature03288 15729348 PMC2175124

[B14] Hemington K. S. Wu Q. Kucyi A. Inman R. D. Davis K. D. (2016). Abnormal cross-network functional connectivity in chronic pain and its association with clinical symptoms. *Brain Struct. Funct.* 221 4203–4219. 10.1007/s00429-015-1161-1 26669874

[B15] Jensen K. B. Kosek E. Petzke F. Carville S. Fransson P. Marcus H. (2009). Evidence of dysfunctional pain inhibition in Fibromyalgia reflected in rACC during provoked pain. Pain. 144 95–100. 10.1016/j.pain.2009.03.018 19410366

[B16] Kaplan C. M. Schrepf A. Vatansever D. Larkin T. E. Mawla I. Ichesco E. (2019). Functional and neurochemical disruptions of brain hub topology in chronic pain. Pain. 160 973–983. 10.1097/j.pain.0000000000001480 30763287 PMC6424595

[B17] Kastrati G. Thompson W. H. Schiffler B. Fransson P. Jensen K. B. (2022). Brain network segregation and integration during painful thermal stimulation. *Cereb. Cortex* 10.1093/cercor/bhab464 [Epub ahead of print]. 34997959 PMC9476629

[B18] Langsrud Ø (2003). ANOVA for unbalanced data: use Type II instead of Type III sums of squares. *Stat. Comput.* 13 163–167.

[B19] Larkin T. E. Kaplan C. M. Schrepf A. Ichesco E. Mawla I. Harte S. E. (2021). Altered network architecture of functional brain communities in chronic nociplastic pain. *NeuroImage* 226 117504. 10.1016/j.neuroimage.2020.117504 33293261

[B20] Lee J.-J. Kim H. J. Čeko M. Park B. Lee S. A. Park H. (2021). A neuroimaging biomarker for sustained experimental and clinical pain. *Nat. Med.* 27 174–182. 10.1038/s41591-020-1142-7 33398159 PMC8447264

[B21] Liégeois R. Ziegler E. Phillips C. Geurts P. Gómez F. Bahri M. A. (2016). Cerebral functional connectivity periodically (de)synchronizes with anatomical constraints. *Brain Struct. Funct.* 221 2985–2997. 10.1007/s00429-015-1083-y 26197763

[B22] Mano H. Kotecha G. Leibnitz K. Matsubara T. Sprenger C. Nakae A. (2018). Classification and characterisation of brain network changes in chronic back pain: a multicenter study. *Wellcome Open Res.* 3:19. 10.12688/wellcomeopenres.14069.2 29774244 PMC5930551

[B23] Menon V. Uddin L. Q. (2010). Saliency, switching, attention and control: a network model of insula function. *Brain Struct. Funct.* 214 655–667. 10.1007/s00429-010-0262-0 20512370 PMC2899886

[B24] Napadow V. LaCount L. Park K. As-Sanie S. Clauw D. J. Harris R. E. (2010). Intrinsic brain connectivity in fibromyalgia is associated with chronic pain intensity. *Arthritis Rheum.* 62 2545–2555. 10.1002/art.27497 20506181 PMC2921024

[B25] Nikolaus S. Bode C. Taal E. van de Laar M. A. F. J. (2013). Fatigue and factors related to fatigue in rheumatoid arthritis: a systematic review. *Arthritis Care Res.* 65 1128–1146. 10.1002/acr.21949 23335492

[B26] Power J. D. Mitra A. Laumann T. O. Snyder A. Z. Schlaggar B. L. Petersen S. E. (2014). Methods to detect, characterize, and remove motion artifact in resting state fMRI. *NeuroImage* 84 320–341. 10.1016/j.neuroimage.2013.08.048 23994314 PMC3849338

[B27] Razlighi Q. R. (2018). Task-evoked negative BOLD response in the Default Mode Network does not alter its functional connectivity. *Front. Comput. Neurosci.* 12:67. 10.3389/fncom.2018.00067 30177878 PMC6109759

[B28] Richter C. G. Thompson W. H. Bosman C. A. Fries P. (2015). A jackknife approach to quantifying single-trial correlation between covariance-based metrics undefined on a single-trial basis. *NeuroImage* 114 57–70. 10.1016/j.neuroimage.2015.04.040 25917516

[B29] Sandström A. Ellerbrock I. Jensen K. B. Martinsen S. Altawil R. Hakeberg P. (2019). Altered cerebral pain processing of noxious stimuli from inflamed joints in rheumatoid arthritis: an event-related fMRI study. *Brain Behav. Immun.* 81 272–279. 10.1016/j.bbi.2019.06.024 31228612

[B30] Schaefer A. Kong R. Gordon E. M. Laumann T. O. Zuo X.-N. Holmes A. J. (2018). Local-global parcellation of the human cerebral cortex from intrinsic functional connectivity MRI. *Cereb. Cortex* 28 3095–3114. 10.1093/cercor/bhx179 28981612 PMC6095216

[B31] Seabold S. Perktold J. (2010). “statsmodels: Econometric and statistical modeling with python,” in *Proceedings of the 9th Python in science conference* (Austin, TX: SciPy Society). 10.25080/Majora-92bf1922-011

[B32] Shine J. M. (2019). Neuromodulatory influences on integration and segregation in the brain. *Trends Cogn. Sci.* 23 572–583. 10.1016/j.tics.2019.04.002 31076192

[B33] Shine J. M. Bissett P. G. Bell P. T. Koyejo O. Balsters J. H. Gorgolewski K. J. (2016). The dynamics of functional brain networks: integrated network states during cognitive task performance. *Neuron* 92 544–554. 10.1016/j.neuron.2016.09.018 27693256 PMC5073034

[B34] Sporns O. (2015). Cerebral cartography and connectomics. *Philos. Trans. R. Soc. Lond. B Biol. Sci.* 370:20140173. 10.1098/rstb.2014.0173 25823870 PMC4387514

[B35] Tagliazucchi E. Balenzuela P. Fraiman D. Chialvo D. R. (2010). Brain resting state is disrupted in chronic back pain patients. *Neurosci. Lett.* 485 26–31. 10.1016/j.neulet.2010.08.053 20800649 PMC2954131

[B36] Thompson W. H. Brantefors P. Fransson P. (2017a). From static to temporal network theory: applications to functional brain connectivity. *Netw. Neurosci.* 1 69–99. 10.1162/NETN_a_00011 29911669 PMC5988396

[B37] Thompson W. H. Fanton S. (2021). netplotbrain. *Zenodo* [Preprint]. 10.5281/zenodo.4593837

[B38] Thompson W. H. Kastrati G. Finc K. Wright J. Shine J. M. Poldrack R. A. (2020). Time-varying nodal measures with temporal community structure: a cautionary note to avoid misinterpretation. *Hum. Brain Mapp.* 41 2347–2356. 10.1002/hbm.24950 32058633 PMC7268033

[B39] Thompson W. H. Richter C. G. Plavén-Sigray P. Fransson P. (2017b). A simulation and comparison of dynamic functional connectivity methods. *BioRxiv* [Preprint] 10.1101/212241

[B40] Thompson W. H. Richter C. G. Plaven-Sigray P. Fransson P. (2018). Simulations to benchmark time-varying connectivity methods for fMRI. *PLoS Comput. Biol.* 14:e1006196. 10.1371/journal.pcbi.1006196 29813064 PMC5993323

[B41] Tononi G. (1998). Complexity and coherency: integrating information in the brain. *Trends Cogn. Sci.* 2 474–484. 10.1016/S1364-6613(98)01259-5 21227298

[B42] Uddin L. Q. (2015). Salience processing and insular cortical function and dysfunction. *Nat. Rev. Neurosci.* 16 55–61. 10.1038/nrn3857 25406711

[B43] Xie H. Zheng C. Y. Handwerker D. A. Bandettini P. A. Calhoun V. D. Mitra S. (2019). Efficacy of different dynamic functional connectivity methods to capture cognitively relevant information. *NeuroImage* 188 502–514. 10.1016/j.neuroimage.2018.12.037 30576850 PMC6401299

[B44] Yeo B. T. Krienen F. M. Sepulcre J. Sabuncu M. R. Lashkari D. Hollinshead M. (2011). The organization of the human cerebral cortex estimated by intrinsic functional connectivity. *J. Neurophysiol.* 106 1125–1165. 10.1152/jn.00338.2011 21653723 PMC3174820

