# Supplementary material for: Multiple spatial scale mapping of time-resolved brain network reconfiguration during evoked pain in patients with rheumatoid arthritis
Source: Front Neurosci. 2022 Aug 9;16:942136. doi: 10.3389/fnins.2022.942136 (PMC9397124; doi:10.3389/fnins.2022.942136)
Supplement: Supplementary file 1 [file Data_Sheet_1.DOCX]

Supplementary Material

**fMRIPrep pre-processing pipeline**

Note that the sections on anatomical and functional data pre-processing stated below were automatically generated by the fMRIPrep (Esteban et al., 2019) pre-processing pipeline. We therefore include it here in unaltered form (apart from some very minor stylistic changes), since this is recommended by the developers by fMRIPrep (Esteban et al., 2019).

Results included in this manuscript come from preprocessing performed using fMRIPrep 20.1.1 (Esteban et al., 2019) (RRID:SCR_016216), which is based on Nipype 1.5.0 (Gorgolewski et al., 2011) (RRID:SCR_002502).

**Anatomical data pre-processing**

A total of 1 T1-weighted (T1w) images were found within the input BIDS dataset. The T1-weighted (T1w) image was corrected for intensity non-uniformity (INU) with N4BiasFieldCorrection (Tustison et al., 2010), distributed with ANTs 2.2.0 (Avants et al., 2008) (RRID:SCR_004757), and used as T1w-reference throughout the workflow. The T1w-reference was then skull-stripped with a Nipype implementation of the antsBrainExtraction.sh workflow (from ANTs), using OASIS30ANTs as target template. Brain tissue segmentation of cerebrospinal fluid (CSF), white-matter (WM) and gray-matter (GM) was performed on the brain-extracted T1w using fast (Zhang et al., 2001) (FSL 5.0.9, RRID:SCR_002823).

Brain surfaces were reconstructed using recon-all (Dale et al., 1999) (FreeSurfer 6.0.1, RRID:SCR_001847), and the brain mask estimated previously was refined with a custom variation of the method to reconcile ANTs-derived and FreeSurfer-derived segmentations of the cortical gray-matter of Mindboggle (Klein et al., 2017) (RRID:SCR_002438). Volume-based spatial normalization to one standard space (MNI152NLin2009cAsym) was performed through nonlinear registration with antsRegistration (ANTs 2.2.0), using brain-extracted versions of both T1w reference and the T1w template. The following template was selected for spatial normalization: ICBM 152 Nonlinear Asymmetrical template version 2009c (Fonov et al., 2009) (RRID:SCR_008796; TemplateFlow ID: MNI152NLin2009cAsym).

**Functional data pre-processing**

For each of the 4 BOLD runs found per subject (across all tasks and sessions), the following preprocessing was performed. First, a reference volume and its skull-stripped version were generated using a custom methodology of fMRIPrep. Head-motion parameters with respect to the BOLD reference (transformation matrices, and six corresponding rotation and translation parameters) are estimated before any spatiotemporal filtering using mcflirt (Jenkinson et al., 2002) (FSL 5.0.9). Susceptibility distortion correction (SDC) was omitted. The BOLD reference was then co-registered to the T1w reference using bbregister (FreeSurfer) which implements boundary-based registration (Greve & Fischl, 2009). Co-registration was configured with six degrees of freedom.

The BOLD time-series (including slice-timing correction when applied) were resampled onto their original, native space by applying the transforms to correct for head-motion. These resampled BOLD time-series will be referred to as preprocessed BOLD in original space, or just preprocessed BOLD. The BOLD time-series were resampled into standard space, generating a preprocessed BOLD run in MNI152NLin2009cAsym space.

Several confounding time-series were calculated based on the preprocessed BOLD: framewise displacement (FD), DVARS and three region-wise global signals. FD was computed using two formulations following Power (absolute sum of relative motions, (Power et al., 2014)) and Jenkinson (relative root mean square displacement between affines, (Jenkinson et al., 2002)). FD and DVARS are calculated for each functional run, both using their implementations in Nipype (following the definitions by Power et al. (Power et al., 2014)). The three global signals are extracted within the CSF, the WM, and the whole-brain masks. Additionally, a set of physiological regressors were extracted to allow for component-based noise correction (CompCor) (Behzadi et al., 2007). Principal components are estimated after high-pass filtering the preprocessed BOLD time-series (using a discrete cosine filter with 128s cut-off) for the two CompCor variants: temporal (tCompCor) and anatomical (aCompCor). tCompCor components are then calculated from the top 5% variable voxels within a mask covering the subcortical regions. This subcortical mask is obtained by heavily eroding the brain mask, which ensures it does not include cortical GM regions. For aCompCor, components are calculated within the intersection of the aforementioned mask and the union of CSF and WM masks calculated in T1w space, after their projection to the native space of each functional run (using the inverse BOLD-to-T1w transformation). Components are also calculated separately within the WM and CSF masks. For each CompCor decomposition, the k components with the largest singular values are retained, such that the retained components\u2019 time series are sufficient to explain 50 percent of variance across the nuisance mask (CSF, WM, combined, or temporal). The remaining components are dropped from consideration.

The head-motion estimates calculated in the correction step were also placed within the corresponding confounds file. The confound time series derived from head motion estimates and global signals were expanded with the inclusion of temporal derivatives and quadratic terms for each (Satterthwaite et al., 2013). Frames that exceeded a threshold of 0.5 mm FD or 1.5 standardized DVARS were annotated as motion outliers. All resamplings can be performed with a single interpolation step by composing all the pertinent transformations (i.e. head-motion transform matrices, susceptibility distortion correction when available, and co-registrations to anatomical and output spaces). Gridded (volumetric) resamplings were performed using antsApplyTransforms (ANTs), configured with Lanczos interpolation to minimize the smoothing effects of other kernels (Lanczos, 1964). Non-gridded (surface) resamplings were performed using mri_vol2surf (FreeSurfer).

Many internal operations of fMRIPrep use Nilearn 0.6.2 (Abraham et al., 2014) (RRID:SCR_001362), mostly within the functional processing workflow. For more details of the pipeline, see the section corresponding to workflows in fMRIPrep’s documentation (<https://fmriprep.readthedocs.io/en/latest/workflows.html>).

**Reference List**

Abraham, A., Pedregosa, F., Eickenberg, M., Gervais, P., Mueller, A., Kossaifi, J., Gramfort, A., Thirion, B., & Varoquaux, G. (2014). Machine learning for neuroimaging with scikit-learn. *Frontiers in Neuroinformatics*, *8*. https://doi.org/10.3389/fninf.2014.00014

Avants, B., Epstein, C., Grossman, M., & Gee, J. (2008). Symmetric diffeomorphic image registration with cross-correlation: Evaluating automated labeling of elderly and neurodegenerative brain. *Medical Image Analysis*, *12*(1), 26–41. https://doi.org/10.1016/j.media.2007.06.004

Behzadi, Y., Restom, K., Liau, J., & Liu, T. T. (2007). A component based noise correction method (CompCor) for BOLD and perfusion based fMRI. *NeuroImage*, *37*(1), 90–101. https://doi.org/10.1016/j.neuroimage.2007.04.042

Dale, A. M., Fischl, B., & Sereno, M. I. (1999). *Cortical Surface-Based Analysis: I. Segmentation and Surface Reconstruction*. *9*(2), 179–194.

Esteban, O., Markiewicz, C. J., Blair, R. W., Moodie, C. A., Isik, A. I., Erramuzpe, A., Kent, J. D., Goncalves, M., DuPre, E., Snyder, M., Oya, H., Ghosh, S. S., Wright, J., Durnez, J., Poldrack, R. A., & Gorgolewski, K. J. (2019). fMRIPrep: A robust preprocessing pipeline for functional MRI. *Nature Methods*, *16*(1), 111–116. https://doi.org/10.1038/s41592-018-0235-4

Fonov, V., Evans, A., McKinstry, R., Almli, C., & Collins, D. (2009). *Unbiased nonlinear average age-appropriate brain templates from birth to adulthood*. *47*, S102.

Gorgolewski, K., Burns, C. D., Madison, C., Clark, D., Halchenko, Y. O., Waskom, M. L., & Ghosh, S. S. (2011). Nipype: A Flexible, Lightweight and Extensible Neuroimaging Data Processing Framework in Python. *Frontiers in Neuroinformatics*, *5*. https://doi.org/10.3389/fninf.2011.00013

Greve, D. N., & Fischl, B. (2009). Accurate and robust brain image alignment using boundary-based registration. *NeuroImage*, *48*(1), 63–72. https://doi.org/10.1016/j.neuroimage.2009.06.060

Jenkinson, M., Bannister, P., Brady, M., & Smith, S. (2002). Improved Optimization for the Robust and Accurate Linear Registration and Motion Correction of Brain Images. *NeuroImage*, *17*(2), 825–841. https://doi.org/10.1006/nimg.2002.1132

Klein, A., Ghosh, S. S., Bao, F. S., Giard, J., Häme, Y., Stavsky, E., Lee, N., Rossa, B., Reuter, M., Chaibub Neto, E., & Keshavan, A. (2017). Mindboggling morphometry of human brains. *PLOS Computational Biology*, *13*(2), e1005350. https://doi.org/10.1371/journal.pcbi.1005350

Lanczos, C. (1964). *Evaluation of Noisy Data*. *1*(1), 76–85.

Power, J. D., Mitra, A., Laumann, T. O., Snyder, A. Z., Schlaggar, B. L., & Petersen, S. E. (2014). Methods to detect, characterize, and remove motion artifact in resting state fMRI. *NeuroImage*, *84*, 320–341. https://doi.org/10.1016/j.neuroimage.2013.08.048

Satterthwaite, T. D., Elliott, M. A., Gerraty, R. T., Ruparel, K., Loughead, J., Calkins, M. E., Eickhoff, S. B., Hakonarson, H., Gur, R. C., Gur, R. E., & Wolf, D. H. (2013). An improved framework for confound regression and filtering for control of motion artifact in the preprocessing of resting-state functional connectivity data. *NeuroImage*, *64*, 240–256. https://doi.org/10.1016/j.neuroimage.2012.08.052

Tustison, N. J., Avants, B. B., Cook, P. A., Yuanjie Zheng, Egan, A., Yushkevich, P. A., & Gee, J. C. (2010). N4ITK: Improved N3 Bias Correction. *IEEE Transactions on Medical Imaging*, *29*(6), 1310–1320. https://doi.org/10.1109/TMI.2010.2046908

Zhang, Y., Brady, M., & Smith, S. (2001). *Segmentation of brain MR images through a hidden Markov random field model and the expectation-maximization algorithm*. *20*(1), 45–57. https://doi.org/10.1109/42.906424

|  | **index** |
| --- | --- |
| **L anterior insula** | 98 |
| **R anterior insula** | 308 |
| **L posterior insula** | 34 |
| **R posterior insula** | 236 |
| **L anterior cingulate gyrus** | 177 |
| **R anterior cingulate gyrus** | 384 |

**Supplementary Table 1.** The six selected nodes and their respective index found on the following TemplateFlow metadata file on <https://www.templateflow.org/browse/>: tpl-MNI152NLin2009cAsym_atlas-Schaefer2018_desc-400Parcels7Networks_dseg.tsv. L, left; R, right.

| **z** | | | | | | | | | | | | |
| --- | --- | --- | --- | --- | --- | --- | --- | --- | --- | --- | --- | --- |
|  | **0** | | | **+1** | | | **+2** | | | **+3** | | |
|  | **F** | **p** | **FDR**  **p** | **F** | **p** | **FDR**  **p** | **F** | **p** | **FDR**  **p** | **F** | **p** | **FDR**  **p** |
| **Cont** | 0.292 | 0.591 | 0.932 | 2.051 | 0.159 | 0.932 | 0.093 | 0.762 | 0.932 | 0.029 | 0.866 | 0.932 |
| **Default** | 0.421 | 0.520 | 0.932 | 0.269 | 0.606 | 0.932 | 0.002 | 0.966 | 0.971 | 0.311 | 0.580 | 0.932 |
| **DorsAttn** | 1.211 | 0.277 | 0.932 | 2.808 | 0.100 | 0.932 | 0.232 | 0.632 | 0.932 | 0.141 | 0.709 | 0.932 |
| **Limbic** | 0.891 | 0.350 | 0.932 | 0.214 | 0.646 | 0.932 | 0.034 | 0.854 | 0.932 | 0.624 | 0.434 | 0.932 |
| **SalVentAttn** | 0.164 | 0.688 | 0.932 | 0.252 | 0.618 | 0.932 | 0.074 | 0.787 | 0.932 | 0.047 | 0.830 | 0.932 |
| **SomMot** | 0.001 | 0.971 | 0.971 | 0.073 | 0.788 | 0.932 | 0.057 | 0.812 | 0.932 | 0.078 | 0.781 | 0.932 |
| **Vis** | 0.307 | 0.582 | 0.932 | 0.033 | 0.857 | 0.932 | 0.106 | 0.746 | 0.932 | 0.030 | 0.864 | 0.932 |

**Supplementary Table 2.** Results from the analysis of covariance (ANCOVA) computed at the community level on within-module degree z-score (z) values per time point across groups (patients and controls) when pain was delivered to the diseased joint in patients and corresponding site in controls. Time points are indicated as 0, +1, +2, +3 and represent, respectively, the onset TR (TR=3s) of painful stimulation, and +1, +2, and +3 TR after-stimulation. FDR, false discovery rate.


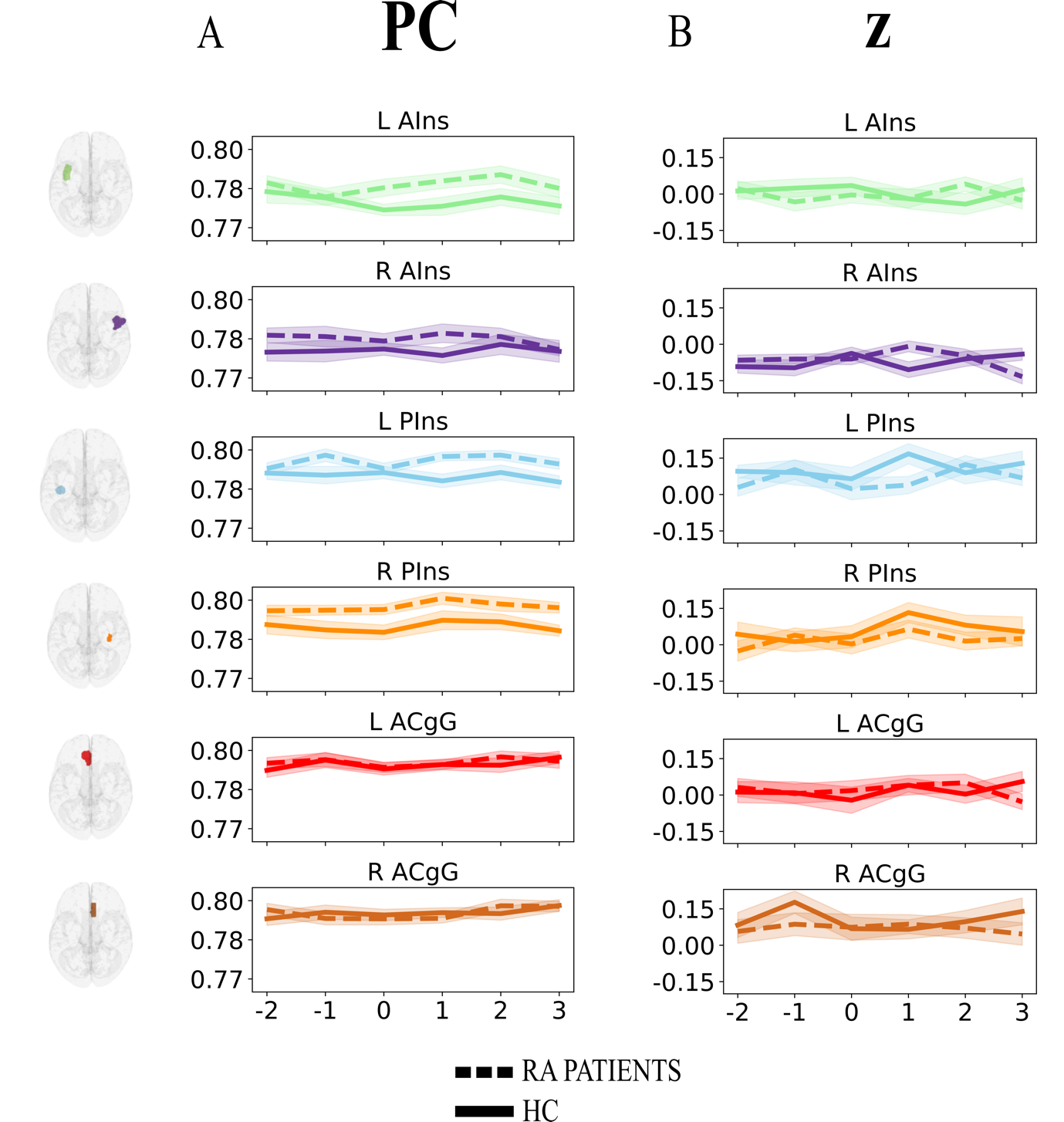


**Supplementary Figure 1.** Degree of change in node-related integration (A) and segregation (B) per time point across rheumatoid arthritis (RA) patients and healthy controls (HC), when pain was delivered to the diseased joint and corresponding site in HC. Displayed are the average parameter values (PC, z) over trials per time point. Time points are indicated as -2, -1, 0, +1, +2, +3 on the x-axis, with 0 representing the onset TR (TR=3s) of painful stimulation, -2 and -1 being the two TR pre-stimulation, whereas +1, +2, and +3 the three TR after-stimulation. The brain plots on the left side of the figure represent each of the six nodes selected to represent key chronic pain brain regions (see below for a complete list). The shaded areas contouring the lines represent the standard error of the mean. PC, participation coefficient; z, within-module degree z-score; L AIns, left anterior insula; R AIns, right anterior insula; L PIns, left posterior insula; R PIns, right posterior insula; L ACgG, left anterior cingulate gyrus; R ACgG, right anterior cingulate gyrus.

| **PC** | | | | | | | | | | | | |
| --- | --- | --- | --- | --- | --- | --- | --- | --- | --- | --- | --- | --- |
|  | **0** | | | **+1** | | | **+2** | | | **+3** | | |
|  | **F** | **p** | **FDR**  **p** | **F** | **p** | **FDR**  **p** | **F** | **p** | **FDR**  **p** | **F** | **p** | **FDR**  **p** |
| **L AIns** | 2.863 | 0.097 | 0.233 | 4.722 | 0.035 | 0.137 | 3.991 | 0.052 | 0.137 | 2.153 | 0.149 | 0.306 |
| **R AIns** | 0.454 | 0.504 | 0.719 | 2.108 | 0.153 | 0.306 | 0.564 | 0.456 | 0.719 | 0.017 | 0.898 | 0.937 |
| **L PIns** | 0.209 | 0.650 | 0.793 | 5.166 | 0.028 | 0.137 | 4.615 | 0.037 | 0.137 | 5.232 | 0.027 | 0.137 |
| **R PIns** | 4.634 | 0.037 | 0.137 | 5.215 | 0.027 | 0.137 | 4.187 | 0.046 | 0.137 | 4.440 | 0.040 | 0.137 |
| **L ACgG** | 0.022 | 0.883 | 0.937 | 0.002 | 0.962 | 0.962 | 1.080 | 0.304 | 0.561 | 0.383 | 0.539 | 0.719 |
| **R ACgG** | 0.195 | 0.661 | 0.793 | 0.383 | 0.539 | 0.719 | 0.855 | 0.360 | 0.617 | 0.035 | 0.853 | 0.937 |

**Supplementary Table 3.** Results from the analysis of covariance (ANCOVA) computed at the nodal level on participation coefficient (PC) values per time point across groups (patients and controls) when pain was delivered to the diseased joint in patients and corresponding site in controls. Time points are indicated as 0, +1, +2, +3 and represent, respectively, the onset TR (TR=3s) of painful stimulation, and +1, +2, and +3 TR after-stimulation. FDR, false discovery rate.

| **z** | | | | | | | | | | | | |
| --- | --- | --- | --- | --- | --- | --- | --- | --- | --- | --- | --- | --- |
|  | **0** | | | **+1** | | | **+2** | | | **+3** | | |
|  | **F** | **p** | **FDR**  **p** | **F** | **p** | **FDR**  **p** | **F** | **p** | **FDR**  **p** | **F** | **p** | **FDR**  **p** |
| **L AIns** | 0.695 | 0.409 | 0.780 | 0.000 | 0.100 | 1.000 | 2.887 | 0.096 | 0.445 | 0.761 | 0.387 | 0.780 |
| **R AIns** | 0.490 | 0.487 | 0.780 | 6.790 | 0.012 | 0.155 | 0.122 | 0.729 | 0.874 | 6.679 | 0.013 | 0.155 |
| **L PIns** | 0.733 | 0.396 | 0.780 | 3.890 | 0.054 | 0.436 | 0.227 | 0.636 | 0.852 | 1.267 | 0.266 | 0.709 |
| **R PIns** | 0.223 | 0.639 | 0.852 | 1.941 | 0.170 | 0.583 | 1.442 | 0.236 | 0.708 | 0.254 | 0.616 | 0.852 |
| **L ACgG** | 0.505 | 0.481 | 0.780 | 0.000 | 0.990 | 1.000 | 0.526 | 0.472 | 0.780 | 2.731 | 0.105 | 0.445 |
| **R ACgG** | 0.005 | 0.941 | 1.000 | 0.082 | 0.776 | 0.887 | 0.143 | 0.707 | 0.874 | 2.635 | 0.111 | 0.445 |

**Supplementary Table 4.** Results from the analysis of covariance (ANCOVA) computed at the nodal level on within-module degree z-score (z) values per time point across groups (patients and controls) when pain was delivered to the diseased joint in patients and corresponding site in controls. Time points are indicated as 0, +1, +2, +3 and represent, respectively, the onset TR (TR=3s) of painful stimulation, and +1, +2, and +3 TR after-stimulation. FDR, false discovery rate.


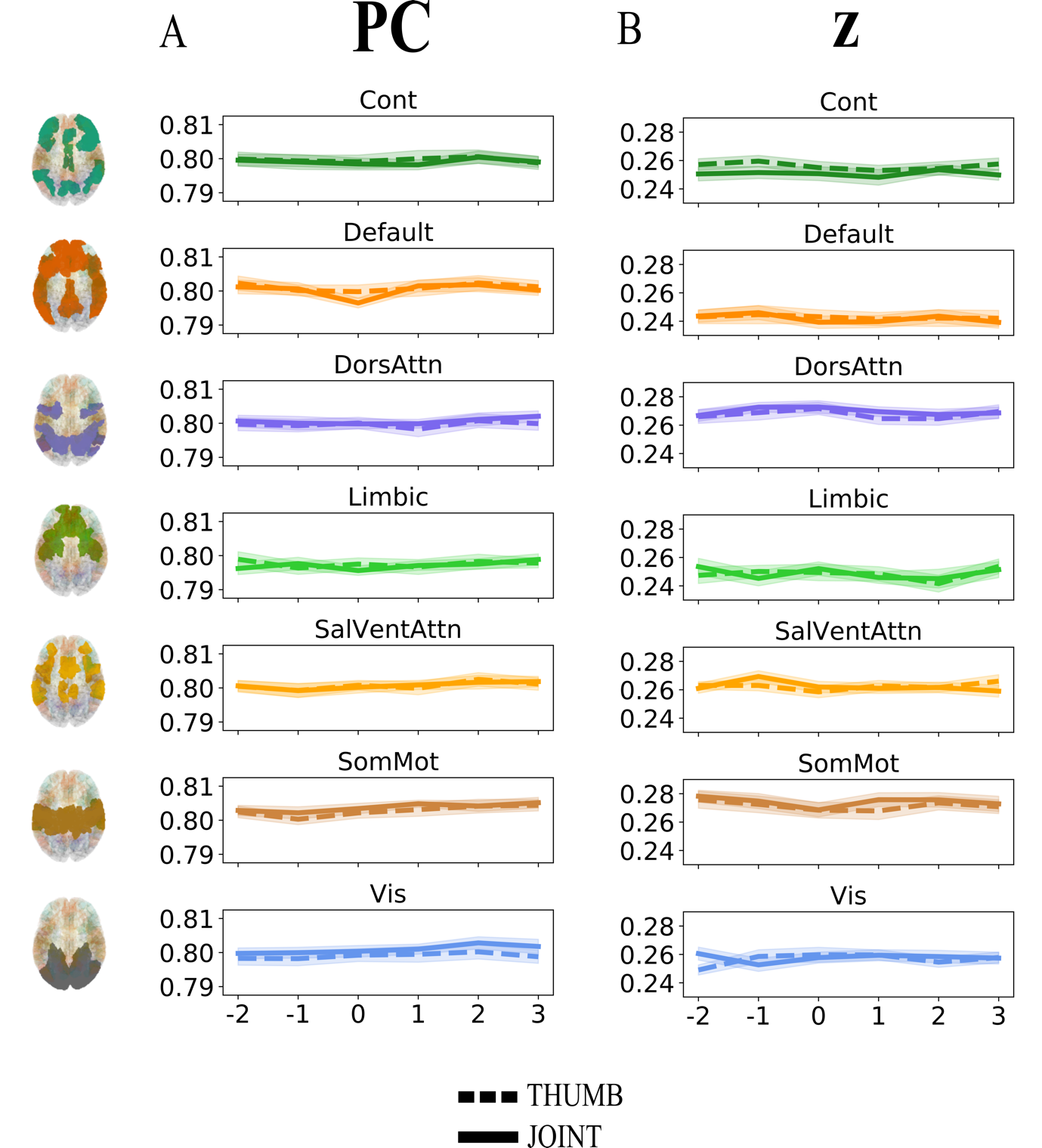


**Supplementary Figure 2.** Degree of change in community-related integration (A) and segregation (B) per time point when pain was delivered to the diseased joint compared to the neutral thumb in rheumatoid arthritis (RA) patients. Displayed are the average parameter values (PC, z) over trials per time point. Time points are indicated as -2, -1, 0, +1, +2, +3 on the x-axis, with 0 representing the onset TR (TR=3s) of painful stimulation, -2 and -1 being the two TR pre-stimulation, whereas +1, +2, and +3 the three TR after-stimulation. The brain plots on the left side of the figure represent each of the seven Yeo communities (Yeo et al., 2011). The shaded areas contouring the lines represent the standard error of the mean. PC, participation coefficient; z, within-module degree z-score.


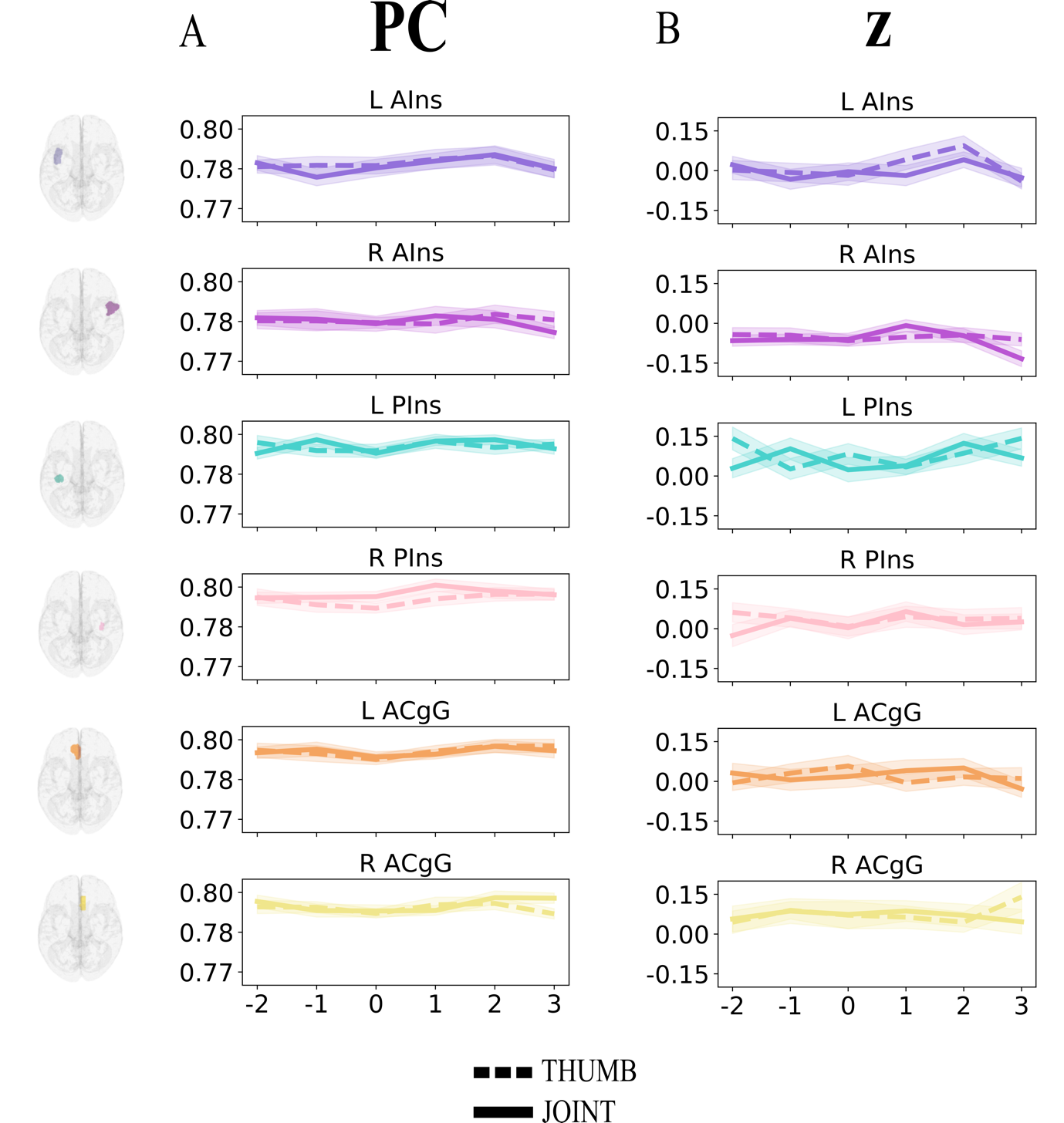


**Supplementary Figure 3.** Degree of change in node-related integration (A) and segregation (B) per time point when pain was delivered to the diseased joint compared to the neutral thumb in rheumatoid arthritis (RA) patients. Displayed are the average parameter values (PC, z) over trials per time point. Time points are indicated as -2, -1, 0, +1, +2, +3 on the x-axis, with 0 representing the onset TR (TR=3s) of painful stimulation, -2 and -1 being the two TR pre-stimulation, whereas +1, +2, and +3 the three TR after-stimulation. The brain plots on the left side of the figure represent each of the six nodes selected to represent key chronic pain brain regions (see below for a complete list). The shaded areas contouring the lines represent the standard error of the mean. PC, participation coefficient; z, within-module degree z-score; L AIns, left anterior insula; R AIns, right anterior insula; L PIns, left posterior insula; R PIns, right posterior insula; L ACgG, left anterior cingulate gyrus; R ACgG, right anterior cingulate gyrus.

| **PC** | | | | | | | | | | | | |
| --- | --- | --- | --- | --- | --- | --- | --- | --- | --- | --- | --- | --- |
|  | **0** | | | **+1** | | | **+2** | | | **+3** | | |
|  | **ß** | **p** | **FDR**  **p** | **ß** | **p** | **FDR**  **p** | **ß** | **p** | **FDR**  **p** | **ß** | **p** | **FDR**  **p** |
| **Cont** | -0.001 | 0.754 | 0.966 | -0.001 | 0.417 | 0.966 | -0.000 | 0.932 | 0.967 | 0.000 | 0.890 | 0.966 |
| **Default** | -0.003 | 0.145 | 0.966 | 0.001 | 0.686 | 0.966 | -0.000 | 0.804 | 0.966 | -0.001 | 0.702 | 0.966 |
| **DorsAttn** | -0.000 | 0.897 | 0.966 | 0.001 | 0.436 | 0.966 | 0.000 | 0.815 | 0.966 | 0.002 | 0.284 | 0.966 |
| **Limbic** | -0.002 | 0.361 | 0.966 | 0.000 | 0.843 | 0.966 | -0.001 | 0.690 | 0.966 | 0.001 | 0.659 | 0.966 |
| **SalVentAttn** | -0.000 | 0.767 | 0.966 | 0.001 | 0.659 | 0.966 | -0.001 | 0.638 | 0.966 | 0.001 | 0.722 | 0.966 |
| **SomMot** | 0.001 | 0.004 | 0.966 | 0.001 | 0.397 | 0.966 | 0.000 | 0.982 | 0.982 | 0.001 | 0.720 | 0.966 |
| **Vis** | 0.001 | 0.554 | 0.966 | 0.001 | 0.423 | 0.966 | 0.002 | 0.219 | 0.966 | 0.002 | 0.132 | 0.966 |

**Supplementary Table 5.** Results from the linear mixed-effects model analysis computed at the community level on participation coefficient (PC) values per time point when pain was delivered to the diseased joint compared to the neutral thumb in patients. Time points are indicated as 0, +1, +2, +3 and represent, respectively, the onset TR (TR=3s) of painful stimulation, and +1, +2, and +3 TR after-stimulation. FDR, false discovery rate.

| **z** | | | | | | | | | | | | |
| --- | --- | --- | --- | --- | --- | --- | --- | --- | --- | --- | --- | --- |
|  | **0** | | | **+1** | | | **+2** | | | **+3** | | |
|  | **ß** | **p** | **FDR**  **p** | **ß** | **p** | **FDR**  **p** | **ß** | **p** | **FDR**  **p** | **ß** | **p** | **FDR**  **p** |
| **Cont** | -0.004 | 0.296 | 0.985 | -0.005 | 0.220 | 0.985 | -0.001 | 0.855 | 0.985 | -0.008 | 0.117 | 0.985 |
| **Default** | -0.004 | 0.395 | 0.985 | -0.002 | 0.683 | 0.985 | 0.001 | 0.769 | 0.985 | -0.003 | 0.459 | 0.985 |
| **DorsAttn** | 0.001 | 0.821 | 0.985 | 0.005 | 0.266 | 0.985 | 0.003 | 0.475 | 0.985 | -0.001 | 0.839 | 0.985 |
| **Limbic** | 0.003 | 0.649 | 0.985 | -0.003 | 0.690 | 0.985 | 0.004 | 0.559 | 0.985 | -0.002 | 0.719 | 0.985 |
| **SalVentAttn** | 0.003 | 0.388 | 0.985 | -0.002 | 0.664 | 0.985 | 0.000 | 0.985 | 0.985 | -0.007 | 0.139 | 0.985 |
| **SomMot** | 0.000 | 0.963 | 0.985 | 0.008 | 0.054 | 0.985 | 0.002 | 0.638 | 0.985 | 0.002 | 0.617 | 0.985 |
| **Vis** | -0.002 | 0.633 | 0.985 | -0.000 | 0.963 | 0.985 | 0.004 | 0.439 | 0.985 | -0.000 | 0.920 | 0.985 |

**Supplementary Table 6.** Results from the linear mixed-effects model analysis computed at the community level on within-module degree z-score (z) values per time point when pain was delivered to the diseased joint compared to the neutral thumb in patients. Time points are indicated as 0, +1, +2, +3 and represent, respectively, the onset TR (TR=3s) of painful stimulation, and +1, +2, and +3 TR after-stimulation. FDR, false discovery rate.

| **PC** | | | | | | | | | | | | |
| --- | --- | --- | --- | --- | --- | --- | --- | --- | --- | --- | --- | --- |
|  | **0** | | | **+1** | | | **+2** | | | **+3** | | |
|  | **ß** | **p** | **FDR**  **p** | **ß** | **p** | **FDR**  **p** | **ß** | **p** | **FDR**  **p** | **ß** | **p** | **FDR**  **p** |
| **L AIns** | -0.001 | 0.807 | 0.978 | -0.001 | 0.882 | 0.978 | 0.000 | 0.908 | 0.978 | 0.000 | 0.888 | 0.978 |
| **R AIns** | -0.000 | 0.896 | 0.978 | 0.003 | 0.515 | 0.978 | -0.002 | 0.580 | 0.978 | -0.005 | 0.274 | 0.978 |
| **L PIns** | -0.001 | 0.735 | 0.978 | 0.000 | 0.978 | 0.978 | 0.003 | 0.258 | 0.978 | -0.002 | 0.574 | 0.978 |
| **R PIns** | 0.004 | 0.187 | 0.978 | 0.005 | 0.095 | 0.978 | 0.001 | 0.626 | 0.978 | -0.000 | 0.937 | 0.978 |
| **L ACgG** | 0.001 | 0.755 | 0.978 | -0.001 | 0.722 | 0.978 | -0.000 | 0.963 | 0.978 | -0.002 | 0.443 | 0.978 |
| **R ACgG** | -0.002 | 0.746 | 0.978 | 0.002 | 0.427 | 0.978 | 0.002 | 0.379 | 0.978 | 0.006 | 0.060 | 0.978 |

**Supplementary Table 7.** Results from the linear mixed-effects model analysis computed at the nodal level on participation coefficient (PC) values per time point when pain was delivered to the diseased joint compared to the neutral thumb in patients. Time points are indicated as 0, +1, +2, +3 and represent, respectively, the onset TR (TR=3s) of painful stimulation, and +1, +2, and +3 TR after-stimulation. FDR, false discovery rate.

| **z** | | | | | | | | | | | | |
| --- | --- | --- | --- | --- | --- | --- | --- | --- | --- | --- | --- | --- |
|  | **0** | | | **+1** | | | **+2** | | | **+3** | | |
|  | **ß** | **p** | **FDR**  **p** | **ß** | **p** | **FDR**  **p** | **ß** | **p** | **FDR**  **p** | **ß** | **p** | **FDR**  **p** |
| **L AIns** | 0.014 | 0.735 | 0.961 | -0.060 | 0.246 | 0.882 | -0.053 | 0.235 | 0.882 | 0.013 | 0.784 | 0.961 |
| **R AIns** | 0.005 | 0.878 | 0.961 | 0.043 | 0.210 | 0.882 | -0.003 | 0.929 | 0.961 | -0.073 | 0.015 | 0.360 |
| **L PIns** | -0.060 | 0.294 | 0.882 | 0.005 | 0.920 | 0.961 | 0.038 | 0.386 | 0.926 | -0.074 | 0.068 | 0.712 |
| **R PIns** | -0.005 | 0.924 | 0.961 | 0.020 | 0.654 | 0.961 | -0.021 | 0.673 | 0.961 | -0.014 | 0.750 | 0.961 |
| **L ACgG** | -0.040 | 0.331 | 0.883 | 0.046 | 0.275 | 0.882 | 0.034 | 0.506 | 0.961 | -0.039 | 0.429 | 0.936 |
| **R ACgG** | 0.003 | 0.961 | 0.961 | 0.023 | 0.637 | 0.961 | 0.026 | 0.696 | 0.961 | -0.093 | 0.089 | 0.712 |

**Supplementary Table 8.** Results from the linear mixed-effects model analysis computed at the nodal level on within-module degree z-score (z) values per time point when pain was delivered to the diseased joint compared to the neutral thumb in patients. Time points are indicated as 0, +1, +2, +3 and represent, respectively, the onset TR (TR=3s) of painful stimulation, and +1, +2, and +3 TR after-stimulation. FDR, false discovery rate.
